# Evaluation of WHO Criteria for Viral Failure in Patients on Antiretroviral Treatment in Resource-Limited Settings

**DOI:** 10.1155/2011/736938

**Published:** 2011-04-10

**Authors:** Barbara Castelnuovo, Joseph Sempa, Kiragga N. Agnes, Moses R. Kamya, Yukari C. Manabe

**Affiliations:** ^1^Infectious Diseases Institute, Makerere University, Mulago Hospital Complex, P.O. Box 22418, Kampala, Uganda; ^2^Department of Medicine, Makerere University College of Health Sciences, Kampala, Uganda; ^3^Division of Infectious Diseases, Department of Medicine, Johns Hopkins University School of Medicine, Baltimore, MD 21205, USA

## Abstract

Our objective was to evaluate outcomes in patients with sustained viral suppression compared to those with episodes of viremia. *Methods*. In a prospective cohort of patients started on ART in Uganda and followed for 48 months, patients were categorized according to viral load (VL): (1) sustained-suppression: (VL ≤1,000 copies/mL) (2) VL 1,001–10,000, or (3) VL >10,000. *Results*. Fifty-Three (11.2%) and 84 (17.8%) patients had a first episode of intermediate and high viremia, respectively. Patients with sustained suppression had better CD4+ T cell count increases over time compared to viremic patients (*P* < .001). The majority of patients with viremia achieved viral suppression when the measurement was repeated. Only 39.6% of patients with intermediate and 19.1% with high viremia eventually needed to be switched to second line (*P* = .008). *Conclusions*. The use of at least one repeat measurement rather than a single VL measurement could avert from 60% to 80% of unnecessary switches.

## 1. Introduction

Data from developed countries suggest that episodes of low (400–1000 copies/mL) and transient viremia while on antiretroviral treatment (ART) have limited consequences on patients' clinical and immunological outcomes and have a low risk of developing drug resistance [1–4], while episodes of viremia >1,000 copies/mL have been associated with new clinical events [5]. The optimal threshold at which patients should be switched to second line is still debated. It has been reported that patients may not have detrimental effects on CD4 T-cell counts [6] and clinical progression of disease [7] despite having detectable viral load (VL) above 10,000 copies/mL. The 2006 World Health Organization (WHO) guidelines recommended switching patients to second-line ART if the VL was >10,000 copies/mL. Therefore, patients with VL between 1000 and 10,000 copies/mL were usually maintained on first-line treatment and no specific guidance was available to clinicians [8]. Recently the WHO published a document [9] recommending that patients with a VL >5,000 copies/mL should have a VL repeated and, if the VL remains >5,000 copies/mL, should prompt therapy change. 

The objective of our study was to evaluate clinical, immunological, virological, and therapeutic outcomes in patients with sustained viral suppression compared to patients with first episodes of viremia (categorized by magnitude) after at least 6 months of ART.

## 2. Methods

We analyzed data from a prospective cohort of patients started on first-line ART between April 2004 and April 2005 and followed for 4 years at the Infectious Diseases Institute, Kampala, Uganda. Patients were started on stavudine, lamivudine, and nevirapine (provided by Global Fund), or on zidovudine plus lamivudine plus efavirenz (provided by the US President's Emergency Plan for AIDS Relief).

A detailed description of the study has been published elsewhere [10]. Briefly, the study subjects are assessed clinically every 3 months and laboratory testing including CD4+ T-cell count by FACS Count (Becton Dickinson, Mountain View, California, USA), and HIV-1 VL (Amplicor HIV-1 Monitor PCR Test, version 1.5, Roche Diagnostic, GmbH Molecular Systems, Pleasanton, California, USA), with a detection limit of 400 copies/mL is performed every 6 months. The study was approved by the Institutional Review Board of Makerere University Faculty of Medicine and the Uganda National Council for Science and Technology (no: MV 853). 

Patients were included in the analysis if they had reached 6 months of followup on ART. They were categorized according to VL measurements: (1) subjects with sustained suppression: (VL ≤1,000 copies/mL at each measurement), (2) subjects with a first episode of intermediate viremia (VL between 1,001–10,000 copies/mL), or (3) subjects with a first episode of high viremia (VL >10,000 copies/mL). We also recategorized patients with a first episode of viremia between 1,000–5,000 copies/mL and those with a first episode of viremia >5,000 copies/mL for a second analysis according to the new WHO recommendation. 

Clinical outcome was defined as the occurrence of new opportunistic infections or death after reaching a first episode of viremia. Immunological outcome was defined as the median increase in CD4+ T-cell count at the end of the study and the cumulative probability of reaching a CD4+ T-cell count of 200 cells/*μ*L in patients that had not reached 200 cell/*μ*L at month 6. The virological outcome was defined as the proportions of patients with a consecutive subsequent VL ≤1,000, 1,000–10,000 and >10,000 copies/mL. Finally, the therapeutic outcome was defined as the proportion of patients switched to second line. The standard operating procedure in our clinic for patients with episodes of viremia is to switch patient with 2 consecutive VL >1,000 copies/mL. However, in clinical practice patients are often not switched after 2 consecutive VLs >1,000 copies/mL. This is due to a variety of reasons: clinician reluctance to switch patients due to the lack of subsequent treatment options, subsequent low measures (<10,000) of detectable VLs, and finally irregular supply of the second-line drugs.

### 2.1. Statistical Analysis

We compared the baseline characteristics of patients in different VL groups. We compared baseline characteristics using the Kruskal Wallis test for continuous variables (age, body mass index, hemoglobin, CD4+ count) and chi-square for categorical variables (gender, WHO staging, ART regimen).

We used the Kruskal Wallis test to compare median CD4+ T-cell count increase at followup, chi-square tests to compare proportion of outcomes and proportion of patients that did not achieve a CD4+ T-cell count number >200 cell/*μ*L at month 6, and Kaplan Maier curves to describe the probability of reaching a CD4+ T cell count >200 cell/*μ*L in patients with a CD4+ T cell count ≤200 cell/*μ*L after 6 months of followup.

## 3. Results

Of the 559 patients enrolled in the study, 474 reached at least 6 months on ART; of these patients, one patient had no VL measurement available after 6 months on ART, and therefore, 473 (84.6%) were included the analysis. Sixty-seven died [11], 13 were lost to followup, 4 were transferred other facilities, and 1 withdrew consent before reaching 6 months on ART. The patients were followed up for a median time of 48 months (range 6–48).

### 3.1. Baseline Characteristics and Patients Classification

The majority of the patients (*n* = 336, 71%) had sustained suppression throughout the study, 53 (11.2%) patients had a first episode of intermediate viremia (1,000–10,000 copies/mL) after a median time of 40 weeks (IQR 26–74) and 84 (17.8%) patients had a first episode of high viremia (>10,000 copies/mL) after a median time of 48 weeks (IQR 28–74) (*P* = .624).

The baseline characteristics were similar in the three groups except that a higher proportion (90.6%) of patients with a first episode of intermediate viremia were started on an nevirapine-based regimen as compared to patients with sustained suppression (71.7%) and patients with a first episode of high viremia (72.6%) (*P* = .009) (Table 1).

### 3.2. Opportunistic Infections and Death

We did not observe differences in the proportion of deaths in patients with sustained suppression (6.9%) compared to patients with a first episode of intermediate (5.7%) and high viremia (11.9%) (*P* = .25). 

The proportion of patients experiencing at least one WHO grade 3 or 4 clinical event after 6 months of ART was similar in the three groups (21.1% (sustained), 28.2% (intermediate viremia), and 24.5% (high viremia) *P* = .4). A similar proportion of patients with a first episode of intermediate and high viremia experienced an opportunistic infection before (22.6% versus 27.4%,  *P* = .53) and after the episode of viremia (7.5% versus 8.3%, *P* = .866).

### 3.3. Immunologic Response

**Figure 1:**
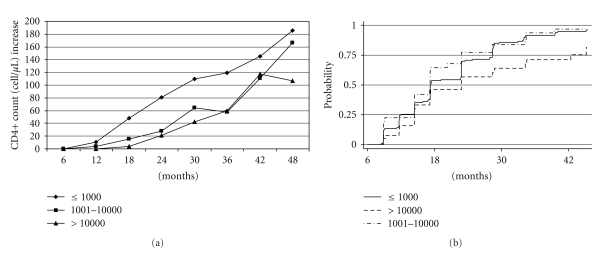
(a) Median increase in CD4+ T-cell count in patient with sustained suppression (viral load VL ≤1,000 copies/mL at each measurement), patients with a first episode of intermediate viremia (viral load between 1,001–10,000 copies/mL), and high viremia (viral load >10,000 copies/mL) over time. (b) Probability of achieving CD4+ T-cell count >200 cells/*μ*L in patient with sustained suppression (viral load VL ≤1,000 copies/mL at each measurement), patients with a first episode of intermediate viremia (viral load between 1,001–10,000 copies/mL, and high viremia (viral load >10,000 copies/mL) over time.

The median CD4+ T-cell count increase at different study intervals was higher in patients with sustained suppression compared to those patients with intermediate and high viremia (*P* = .001) with a total increase at year 4 (or at the last available observation) of 186 cells/*μ*L, 167 cells/*μ*L, and 107 cells/*μ*L, resp. (Figure 1(a)).

Overall, 220 (46.4%) patients did not achieve a CD4+ T-cell count >200 cells/*μ*L at month 6. Only 42.9% (144/336) of those patients who achieved sustained suppression did not achieve a CD4+ T-cell count >200 cells/*μ*L, compared to 58.5% (31/53) and 53.6% (39/84) of those with a first episode of intermediate and high viremia, respectively (*P* = .033). In the patients that had not achieved this threshold by month 6, the probability of achieving >200 cells/*μ*L by year 4 was higher in the patients with sustained suppression (86.8%) and the patients with a first episode of intermediate viremia (96.8%) compared to the patients with a first episode of high viremia (55.6%) (*P* = .017) (Figure 1(b)).

### 3.4. Confirmed Viral Failure after the First Episode of Viremia

Of the 137 patients with at least one viremic episode, 15 (10.9%) were excluded from this analysis because their first viremic episode occurred on their last available visit. As shown in Table 2(a), we did not find differences in the proportion of patients with a consecutive subsequent VL ≤1,000 (*n* = 37, 72.5% versus *n* = 41, 57.8%), 1,000–10,000 (*n* = 3, 5.9% versus *n* = 5, 7.0%), and >10,000 (*n* = 11, 21.6% versus *n* = 25, 35.2%) (*P*  value = .166) in patients with a first episode of intermediate and high viremia.

### 3.5. Switch to Second Line

Reassuringly, none of the patients with sustained suppression were switched to second line. Interestingly, a higher proportion of patients with a first episode of intermediate viremia (*n* = 21, 39.6%) as compared to patients with a first episode of high viremia (*n* = 16, 19.1%) were switched to second line treatment (*P* = .008). 

In a subanalysis, we also evaluated the outcomes of 51 (9.1%) patients that had a first episode of viremia between 400 and 1000 copies/mL; clinical and immunological outcomes were similar to patients with sustained viral suppression below 400 copies/mL. only 5 had a subsequent confirmed viral failure.

### 3.6. Recategorizing Patients Using 5,000 Copies/mL as a Cutoff

Thirty-seven of the 473 (7.8%) total patients had a first viremic episode between 1,000 and 5,000 copies/mL after a median time of 60 weeks (IQR: 26–96) and 85 (18%) patients had a first viremic episode >5,000 copies/mL after a median time of 39 weeks (IQR: 25–74) (*P* = .331). 

As expected, we found no differences in the proportion of patients who died or developed opportunistic infections across the 3 groups, and between patients with first viremic episode between 1,000–5,000 copies/mL and patients with a first viremic episode between 5,000–10,000 copies/mL. 

The median CD4+ T-cell count increase at year 4 (or at the last available observation) was higher in patients with sustained suppression (186 cells/*μ*L) compared to patients with a first viremic episode between 1,001–5,000 copies/mL (167 cells/*μ*L), and patients with a first viremic episode >5,000 copies/mL (100 cells/*μ*L) (*P* < .001). Interestingly, patients with a first episode of viremia between 1,000–5,000 copies/mL had a higher median increase in CD4+ T-cell count as compared to patients with a first episode of viremia between 5,000 and 10,000 copies/mL (167 versus 52 cells/*μ*L) (*P* < .001). In addition, we found that patients with a first viremic episode >5,000 copies/mL had a lower probability of reaching a CD4+ T-cell count of 200 cells/*μ*L as compared to either patients with sustained suppression or patients with a first viremic episode between 1,001–5,000 copies/mL (*P* = .017). However, the probability of reaching a CD4+ T-cell count of 200 cell/*μ*L by year 4 was similar in patients with a first viremic episode between 1,000 and 5,000 and in patients with viremia between 5,000 and 10,000 (*P*  value = .35).

As shown in Table 2(b) a higher proportion of patients with a first viremic episode between 1,001–5,000 copies/mL had a consecutive subsequent VL ≤1,000 copies/mL as compared with patients with viremia >5,000 copies/mL (81.1% versus 55.3%) (*P*  value = .017). 

While the proportion of patients that needed to be switched to second line did not differ statistically (*P* = .293) between the patients with a first episode of viremia between 1,001 and 5,000 copies/mL (33.3%) and those with a first episode of viremia >5,000 copies/mL (24.5%) (*P* = .293).

## 4. Discussion

In our cohort, the majority of the patients (71%) achieved sustained suppression defined as a VL ≤1,000 copies/mL at each visit throughout the study period. Overall, patients with a first episode of intermediate and high viremia do not experience in the medium term (4-year followup) more OIs or deaths as compared to patients that achieve sustained suppression.

Despite the WHO recommendation to switch patients to second line ART if the viral load exceeds 10,000 copies/mL, patients with viremic episodes between 1,001–10,000 copies/mL need to be closely followed because they have an impaired CD4+ T-cell count rise as compared to patients with sustained suppression. Moreover, the long-term consequences of keeping patients on first line with these levels of viremia on morbidity and mortality are not known.

When we analyzed the VL obtained after a first episode of viremia, we found that the majority of the patients with high (72.5%) and intermediate (57.8%) viremia subsequently achieved viral suppression. In our clinic, patients with viral failure receive counseling to re-enforce adherence on their following monthly routine visit, so that VL rebound can be reversed in patients with virus susceptible to the same antiretroviral treatment. 

Conversely, 21.5% of the patients with viremia between 1,001–10,000 copies/mL, who should be kept on first-line therapy according to the 2006 WHO criteria, experienced a subsequent confirmed viral failure. 

In our study, a higher proportion of patients with a first episode of intermediate viremia as compared to ones with a first episode of high viremia were later judged by the clinicians to be in need of second line treatment. An analysis from the UK Collaborative HIV Cohort Study [12] showed that mutations are more frequent in resistance tests performed at VLs between 300 to 10,000 copies/mL and decrease at VLs above 10,000. It is likely that first episodes of very high levels of viremia in our cohort occurred in patients that had discontinued medications without clinicians' knowledge and that, in the long run, manage to achieve suppression after adherence counseling. On the other hand, a first presentation with intermediate viremia could be a sign of emerging drug resistance and, therefore, these patients are likely to experience a subsequent detectable viral load and increasing VL over time. 

Because of the new WHO recommendations [9], we also analyzed the treatment outcomes using a cutoff of 5,000 copies/mL. Patients with a first episode of viremia between 1,001–5,000 copies/mL do not seem to have an impaired immune reconstitution; moreover, more than 80% of the patients in this group have a subsequent VL ≤1,000 copies. 

Despite the newer guidelines provided by WHO, monitoring ART in resource limited settings is still very challenging. Previous research has shown that the currently proposed criteria for assessing treatment failure using immunologic response in the absence of a VL performs poorly in cohorts from resource limited settings, and researchers are advocating for accessible and cheap VL testing [13–17]. However, the ideal timing for VL testing is unclear. Some suggested strategies are to perform VLs on patients suspected to be failing before switching to second line [18], or as an adherence assessing strategy [19].

Our study suggests that one VL is not enough to make a clinical decision on whether to switch treatment to second line. In this cohort in Uganda, almost 60% of the patients with high viremia (>10,000 copies/mL) will subsequently achieve sustained suppression and only 19% were ultimately judged by the clinicians to be in need of second line. This study shows that the use of subsequent VLs measurement to identify patients failing ART could have averted 60% to 80% of unnecessary switches to second-line treatment. While the new cutoff suggested by the WHO seems to correctly identify patients that can be still kept on first-line, only a minority of patients with a first episode of any level of viremia had a persistently detectable VL that prompted our physicians to switch the patients to second line therapy. 

Our study has some limitations. First, the follow-up time after patients had a first viremic episode may not have been long enough to show the effects on morbidity and mortality of remaining on a first line regimen with detectable viremia. The other limitation of the study is that that we do not perform routine genotype resistance testing on detectable samples; therefore, we do not know the effect on the accumulation of resistance mutations in these patients. 

In conclusion, although the long-term consequences of keeping patients with detectable viremia on first-line therapy on clinical events are not known, in resource-limited settings clinicians must weigh the risk of incurring viral resistance in patients with a detectable VL against the high cost of second line ART [20] and the relatively high proportion of patients in our study who subsequently suppressed with adherence counseling. Strong consideration should be given to adherence counseling after the first detectable viral load and a repeated measurement of VL before switching these patients to a second-line regimen in resource limited settings. 

## Figures and Tables

**Table 1:** Comparison of the baseline characteristics of 473 patients classified in three groups according to their level of viremia.

Patients characteristics	Sustained suppression* 336 (71.0%)	Intermediate viremia* 53 (11.2)	High viremia* 84 (17.8)	*P* value
Female, number (%)	240 (71.4)	30 (56.6)	56 (66.7)	.085
Age (years), median (IQR)	35 (30–42)	34 (30–42)	34 (28–38.5)	.168
CD4+ count median cell/*μ*L (IQR)	107 (35–174)	85 (25–154)	89.5 (26–165)	.246
BMI (Kg/m^2^), median IQR	20.1 (18.3–22.5)	20.6 (18.5–20.5)	20.0 (18–22.5)	.816
WHO Stage 3 and 4, number (%)	291 (86.6)	51 (96.2)	76 (90.5)	.102
Hemoglobin median g/dL (IQR)	11.7 (10.4–13)	11.9 (10.8–13.2)	11.6 (10.5–13)	.792
ART, number (%) Nevirapine	241 (71.7)	48 (90.6)	61 (72.6)	.009
Efavirenz	95 (28.3)	5 (9.4)	23 (27.4)	

*Patients were categorized according to viral load measurements after 6 months on treatment. (1) Sustained suppression. Viral load ≤1,000 copies/mL at each measurement. (2) Subjects with a first episode of intermediate viremia. Viral load between 1,001–10,000 copies/mL. (3) Subjects with a first episode of high viremia. Viral load >10,000 copies/mL.

BMI: body mass index; ART: antiretroviral treatment; IQR: interquartile range.

**Table:** (a)

First episode of viremia (copies/mL)	Number of patients*	Subsequent viral load (copies/mL)			
		≤1,000	1,001–10000	>10000	*P* value
1,001–1,0000	51	37 (72.5)	3 (5.9)	11 (21.6)	.166
>10000	71	41 (57.8)	5 (7.0)	25 (35.2)	

*Viral load was available for 122/137 (89.1%) patients with a first episode of viremia.

**Table:** (b)

First episode of viremia (copies/mL)	Number of patients*	Subsequent viral load (copies/mL)			
		≤1,000	1,000–5,000	>5,000	*P* value
1,001–5,000	37	30 (81.1)	1 (2.7)	6 (16.2)	.017
>5,000	85	47 (55.3)	4 (4.7)	34 (40.0)	

*Viral load was available for 122/137 (89.1%) patients with a first episode of viremia.
